# A Systematic Review of Oropharyngeal Dysphagia Models in Rodents

**DOI:** 10.3390/ijerph18094987

**Published:** 2021-05-07

**Authors:** Han-Na Kim, Ji-Youn Kim

**Affiliations:** 1 Department of Dental Hygiene, College of Health and Medical Sciences, Cheongju University, Cheongju 28503, Korea; hnkim@cju.ac.kr; 2 Department of Dental Hygiene, College of Health Science, Gachon University, Incheon 21936, Korea

**Keywords:** oropharyngeal dysphagia, swallowing difficulty, rats, mice

## Abstract

Oropharyngeal dysphagia is a condition characterized by swallowing difficulty in the mouth and pharynx, which can be due to various factors. Animal models of oropharyngeal dysphagia are essential to confirm the cause-specific symptoms, pathological findings, and the effect of treatment. Recently, various animal models of dysphagia have been reported. The purpose of this review is to organize the rodent models of oropharyngeal dysphagia reported to date. The articles were obtained from Medline, Embase, and the Cochrane library, and selected following the PRISMA guideline. The animal models in which oropharyngeal dysphagia was induced in rats or mice were selected and classified based on the diseases causing oropharyngeal dysphagia. The animal used, method of inducing dysphagia, and screening methods and results were collected from the selected 37 articles. Various rodent models of oropharyngeal dysphagia provide distinctive information on atypical swallowing. Applying and analyzing the treatment in rodent models of dysphagia induced from various causes is an essential process to develop symptom-specific treatments. Therefore, the results of this study provide fundamental and important data for selecting appropriate animal models to study dysphagia.

## 1. Introduction

The occurrence of dysphagia is increasing due to multiple factors. Swallowing is a series of biological processes that passes food via the mouth to the esophagus, while protecting the lower respiratory tract using collaborative control of muscles and several nerves, which take control of the upper respiratory tract. It involves the cranial nerves V, VII, IX, X, and XII [1]. When this swallowing disorder occurs, it precludes normal food intake and leads to inhalation of a small piece of food or fluids, which may impose a risk of complications such as aspiration pneumonitis [2]. This swallowing disorder can potentially develop in the elderly, people with chronic illness, and long-term hospitalized patients. It is usually caused by another health condition, such as neurological disease, brain damage, degenerative disease, head and neck cancer, Parkinson’s disease, and dementia [3]. Generally, it is common in patients with stroke, head and neck cancer, amyotrophic lateral sclerosis (ALS), and Parkinson’s disease (PD). To date, studies on dysphagia in people were mostly related to food intake and nutrition status [4,5]; prognosis of treatment of dysphagia [6,7]; and measurement of the level of dysphagia [8,9]. Dysphagia symptoms can be addressed by targeting improvement because it relates to the underlying disease or conditions, the use of therapeutic strategies to improve swallow function, and the implementation of evidence-based strategies. Although research involving human subjects would provide the most relevant information, not all studies can be conducted on human subjects due to ethical limitations. Therefore, it is necessary to use animal models in physiological and anatomical approaches to understand the neurological and structural causes, and more effectively mitigate these swallowing difficulty symptoms. Commonly, animal models are used in studies on neurological damage, sequela of the disease, and anatomical causes of dysphagia. A number of recent studies have used mice or rats to develop animal models presenting dysphagia similar to that in humans. Experimentally induced dysphagia in rodents has been evaluated using various techniques, in terms of whether it reproduces dysphagia of humans and whether it can be applied to pre-clinical phases, thus replacing dysphagia of humans. Therefore, the purpose of this study was to identify experimental animal models applied to oropharyngeal dysphagia using a systematic review of articles, to confirm the features of each experimental model, and to provide up-to-date information for experimental researchers.

## 2. Methods

### 2.1. Search Criteria

**Figure 1:**
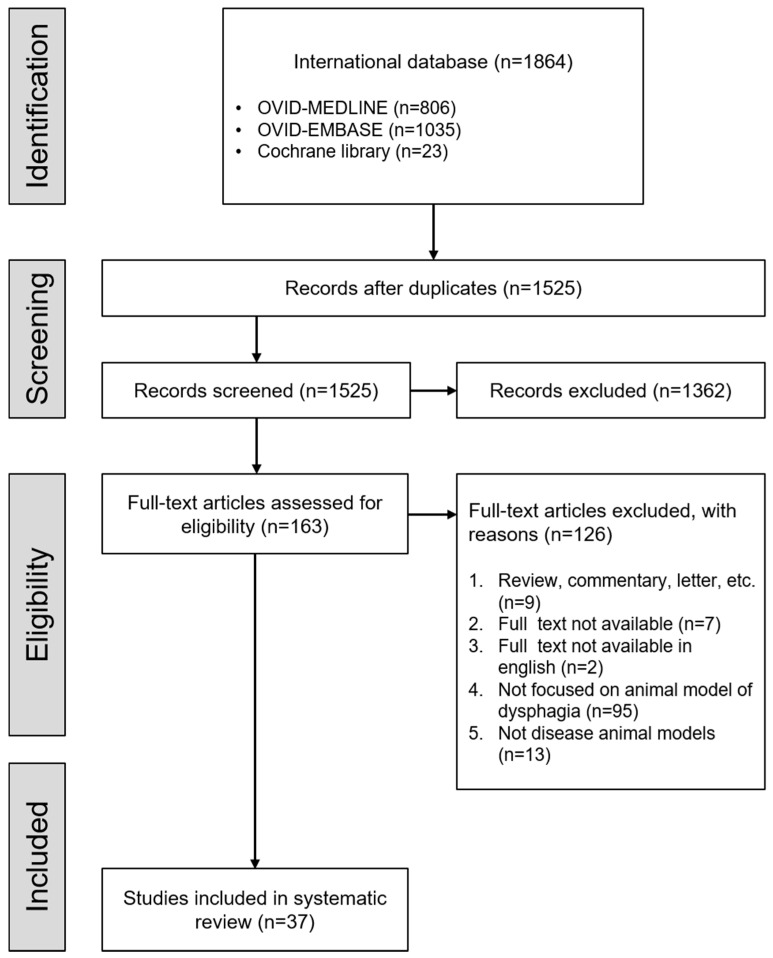
Flowchart of the study selection process.

Following guidelines established by PRISMA [10], the literature search was conducted in March 2020 using Medline, Embase, and the Cochrane library. Our research criteria were established to be specific to experimental studies using animal models of dysphagia. The following Mesh terms were included in the search words: ‘rodent’, ‘mice’, ’rats’, ‘murine’, ‘mus musculus’, ‘deglutition disorder’, ‘dysphagia *’, ‘swallowing disorder *’, ‘swallowing difficult *’, ‘swallowing dysfunction’, and ‘swallowing disease’. There was no restriction on the year of publication. Moreover, only experimental studies using animals were selected. Using the advanced search tools on Medline, Embase, and the Cochrane library, both title and abstract were searched with the following Boolean search strategy: (((((rodent) OR mice) OR rats) OR murine) OR mus musculus) AND (((deglutition disorder) OR dysphagia *) OR (((deglutition) or swallowing) AND ((((disorder) OR disease) OR difficult *) OR dysfunction))). From Medline, 806 articles were given in the final results, and Embase and Cochrane library produced 1035 and 23 articles, respectively. These lists of articles were combined and duplicate references were removed, leaving 1525 articles (Figure 1).

### 2.2. Inclusion/Exclusion Criteria

Title and abstract were screened by J-Y Kim to select the articles for this systematic review. The following inclusion criteria were established: disease animal models inducing oropharyngeal dysphagia and articles that evaluated induced oropharyngeal dysphagia in disease animal models. Aging-related dysphagia were excluded because the purpose was to introduce oropharyngeal dysphagia in disease animal models. Articles using an animal model of dysphagia were excluded unless an evaluation for dysphagia was provided. Only peer-reviewed research articles were included. All other types of articles were excluded.

### 2.3. Assessment of Full-Test Articles

The data from these articles was processed by J-Y Kim and H-N Kim to assess the eligibility criteria defined in the study. To introduce the experimental oropharyngeal dysphagia in rodent models, the following information was considered; disease type, animals, induction protocol of dysphagia, main defects, and dysphagia screening. For disease type, disease models with dysphagia were categorized. Species, sex, and age of animals were recorded. For induction protocol of dysphagia and associated defects, the following information was collected: transgenic animals used, genetically modified animals used, and surgery procedure performed. For the primary defects of disease animal models, symptoms after manipulation were investigated. Finally, for the dysphagia screening, the evaluation methods and results were collected. Although the aim of searching the articles was to report the outcome of any treatment in an animal model of dysphagia, this systematic review focused only on information related to dysphagia.

## 3. Results

Disease rodent models that may cause oropharyngeal dysphagia were verified among the selected 37 articles.

### 3.1. Disease Animal Models

Among rodent models that may cause dysphagia, the number of Parkinson’s disease (PD) models was highest (*n* = 8), followed by stroke (*n* = 7), and amyotrophic lateral sclerosis (ALS) (*n* = 6). Other disease rodent models, such as oculopharyngeal muscular dystrophy (OPMD), Down Syndrome (DS), DiGeorge (22q11) deletion syndrome, Huntington’s disease (HD), multiple sclerosis (MS), Angelman syndrome, cardio-facio-cutaneous syndrome (CFCS), respiratory failure, 5-HT deficiency, cleft palate, and neuro-degenerative disorders were also used.

### 3.2. Parkinson’s Disease (PD)

Table 1 summarizes the characteristics of oropharyngeal dysphagia that were observed in PD rodent models. All of the eight articles used rats as the animal model [11,12,13,14,15,16,17,18]. In most of the articles, rats aged 9 months or younger were used [11,12,14,15,16,17,18], and the age of rats was not presented in one article [13]. Among the eight articles, male rats were used in five articles [11,12,14,15,17], male and female rats in one article [18], and the gender of rats was not mentioned in two articles [13,16]. Among the eight articles, a 6-hydroxydopamine (6-OHDA) rat model of PD via unilateral infusion of 6-OHDA to the medial forebrain bundle was used in five articles [11,12,13,14,15]. PTEN-induced kinase 1 (PINK1) knockout rat, which is a novel model for familial PD, was also used in two articles [16,17]. In the remaining one article, the rat model of PD was created using daily intraperitoneal injections of the rotenone emulsification method [18]. In the three rat models of PD, symptoms such as tail and limb rigidity, akinesia, and bradykinesia were presented; moreover, vocalization and oromotor deficits were observed. To evaluate dysphagia in rat model PD, orolingual motor function analysis such as ultrasonic vocalization analysis, tongue motility, lick assay, and pasta biting were conducted, and videofluorography for deglutition behavior assessment was performed. Based on ultrasonic vocalization analysis, both PINK1^-/-^ and 6-OHDA rat models of PD showed early and progressive vocalization deficits [11,16]. The two types of PD animals showed decreased tongue force, press, and motility while licking [12,14], low bite force, and irregular bite interval; furthermore, it took approximately twice as long to consume a single piece of pasta compared to the control group [13,16]. In addition, in deglutition behavior evaluation, a 6-OHDA rat model of PD had a smaller bolus area that that of the control group [15], and maximum bolus size and velocity were increased and mastication rate was decreased in PINK1^-/-^ rat compared to the control group [17]. Change in jaw movement, chewing cycle, and tongue movement was observed in a rat model of PD by injection with rotenone [18].

### 3.3. Stroke

Table 2 summarizes the characteristics of oropharyngeal dysphagia observed in stroke rodent models. Six to 10 week old or 250–320 g rats were used in five articles, and 18 to 19 week old spontaneous hypertensive rats were used in one article [19,20,21,22,23,24]. In the remaining one article, 7 day old mouse pups were used [25]. An experimental stoke rat model was produced with unilateral or bilateral middle cerebral artery occlusion (MCAO or BCAO) [19,20,21,22,23,24]. The experimental rat models showed symptoms such as focal cerebral ischemia, chronic cerebral hypoperfusion, tongue and licking deficits, and disorders of swallowing reflex. Body weight measurement, neurological assessment, video fluoroscopic swallowing study (VFSS) for swallowing function, electromyogram for swallowing reflex, and bacteria culture were performed to evaluate dysphagia in the stroke model. First, ischemic damage in the ipsilateral external carotid artery (ECA) was verified via MRI [19]. The animals in which ECA ischemic damage was seen showed delayed weight gain and recovery of motor function. In VFSS assessment, the bolus area and speed were decreased in the unilateral MCAO rat model [20]. The number of swallows and the swallowing reflex was decreased in BCAO and MCAO rat models. The onset latency of the first swallow was considerably delayed [21,22,23,24]. Following BCAO, bacterial colonies were reported in the lung culture and the swallowing response was remarkably weakened [23,24]. Mice with cerebral infarct also showed motor disturbance, and it was seen that they were low in weight compared to sham [25].

### 3.4. Amyotrophic Lateral Sclerosis (ALS)

Table 3 summarizes the characteristics of oropharyngeal dysphagia in ALS rodent models. Among a total of six articles on rodent models of ALS, four articles used mice [26,27,28,29] and two articles used rats [30,31]. Articles that used mice all used superoxide dismutase 1 (SOD1)-G93A transgenic mice [26,27,28,29]; in most cases, male and female mice of seven weeks old or younger were used [26,27]. Shock et al. used 4–8 month old transgenic mice models of ALS to compare young healthy mice and aged mice [28]. Among the articles that used rats, one article used male and female SOD1-G93A rats [30]. Another article mimicked dysphagia observed in the ALS rodent model using intralingual injections of cholera toxin B conjugated to saporin (CTB-SAP) into the genioglossus muscle, using 3–4 month old male rats [31]. SOD1-G93A mice or rat models presented dysphagia, which is similar to that of humans with ALS, laryngeal adductor reflux (LAR) impairment, and bulbar motor deficits. Oromotor function analysis, food and water consumption analysis, electrophysiological analysis, laryngoscopy, VFSS, postmortem, and histological analysis were performed to evaluate dysphagia in mice and rat models of ALS. Lever et al. and Smittkamp et al. conducted their study by dividing disease phases into early (maturation), middle (pre-symptomatic), and late (symptomatic and end-stage) in ALS animal models [26,30]. In oromotor function analysis, changes in lick and mastication rates in SOD1-G93A mice were observed in all three phases [26], and a persistent tongue motility deficit was observed in the early phase of ALS [30]. In electrophysiological analysis, it was found that a stronger stimulus to superior laryngeal nerve (SLN) is required for evoking swallowing in SOD1-G93A mice [27]. Through laryngoscopy analysis, it was confirmed that vocal fold (VF) abduction of SOD1-G93A mice was damaged [28]. The change in lick and swallowing pattern in SOD1-G93A mice was detected in VFSS [29]. The weight and size of the tongue were significantly smaller than those of the control group in postmortem analysis. SOD1 mice, having forelimb paralysis and slower lick rates, died at a younger age [29]. Through histological analysis, neurodegeneration of nerves related to mastication and licking behavior, and pharyngeal stage of swallowing, was observed [26]. CTB-SAP treated rats showed death of hypoglossal motor neurons, decreased hypoglossal motor output, and deficit in swallowing and licking [31].

### 3.5. Others

Table 4 summarizes the characteristics of oropharyngeal dysphagia observed in animal models of other diseases. Dysphagia was evaluated in A17 PABPN1 transgenic mice, which are widely used as mouse models of OPMD [32]. Male and female transgenic mice of various ages were used as mouse models of OPMD, and muscle weakness and atrophy were observed. Pharyngeal functions were assessed through lick assay and histological analysis in A17 PANPN1 transgenic mice. For A17 PABPN1 transgenic mice, the growth of the palatopharyngeus muscle was differentially affected depending on its location within the pharynx. Lick rates were decreased with age and impaired. 

Dysphagia was reported in LgDel mice, the animal model of DiGeorge (22q11) deletion syndrome [33,34,35]. In the retrieved articles, littermate mice pups of a month old or younger, or around 3 month old male and female mice, were used. Dysphagia symptoms, and cognitive impairment and craniofacial changes, were presented in LgDel mouse. Body weight measurement, histological analysis, electrophysiological analysis, fluoroscopic and endoscopic assessments, video surveillance, and postmortem analysis were performed in the animal model of DiGeorge (22q11) deletion syndrome to evaluate dysphagia. It was reported that LgDel pups have low weight in the first week after birth, and symptoms of respiratory infections due to food aspirations were presented [33]. Moreover, anomalies in palate and jaw, and alteration of trigeminal (V), glossopharyngeal (IX), or vagus (X) central nerve, which are essential to various feeding and swallowing processes, were observed [33]. The assessment of electrophysiological properties of hypoglossal motor neurons in LgDel mouse p7–14 pups suggested that neurological change in hypoglossal motor neuron function contributes to abnormal swallowing [34]. Fluoroscopic and endoscopic assessments in 3 month old LgDel mice were performed to evaluate swallowing-related structure and function. Consequently, minor structural anomalies of palate and larynx in one-third of LgDel mice were observed [35]. Video surveillance results, in which behaviors related to food intake were assessed, showed that LgDel mice drank and ate more frequently. A considerable amount of lung infection was observed in LgDel mice in postmortem analysis [35]. 

Three published articles reported dysphagia in Ts65Dn and Dp(16)1Yey mice, which are extensively used as mouse models of Down syndrome (DS) [36,37,38]. In one article among the three, 11 day old, male 5–6 week old (young age), and 10–53 week old (adult age) Ts65Dn mice were used in the study [36]. The other two articles used Ts65Dn and Dp(16)1Yey mice: one used 5 week old male mice, whereas the other used 8 to 44 week old male and female mice [37,38]. To evaluate dysphagia in Ts65DS mice, which is the mouse model of DS, molecular biological analysis, such as SDS-PAGE, immunofluorescence, and qRT-PCR, and VFSS and mastication assays for functional feeding and swallowing performance, were undertaken. The result of molecular biological analysis showed that MyHC 2b protein declined in the digastric muscle of 11 day, 5–6 week, and 10–53 week old Ts65Dn mice [36]. In Ts65Dn and Dp(16)1Yey mice, considerable differences in weight, mastication rate, and anterior digastric muscle were shown, and the size of anterior digastric myofibers positive for MyHC 2a were considerably decreased. It was reported that the Ts65Dn model was more impacted than the Dp(16)1Yey model [37]. Changes in swallowing and mastication pattern were presented in the VFSS [38]. 

A transgenic mouse was generated as an animal model of Huntington’s disease (HD) in which Drd1a+ cells were slowly but progressively ablated in the postnatal striatum, yet Drd2 single-positive cells were preserved [39]. Note that male and female mice aged 4 to 18 weeks old were used, and in this HD mouse model, abnormal oral functions of decreased mastication were observed in both male and female mice in the oral behavioral assessment.

Oropharyngeal dysphagia was evaluated in a Human TCR-HLA-DRB1*0401-IA^−/−^ transgenic mouse as an animal model of multiple sclerosis (MS) [40]. Dysphagic symptoms such as tongue paralysis and gait disturbance were observed in this transgenic mouse. Clinical features and histopathological features were analyzed to confirm dysphagia. The result showed that digestive disorders were seen in the transgenic mouse because of restricted motions of jaw and tongue. 

Oropharyngeal dysphagia was reported in the animal model of Angelman syndrome [41]. As the animal model of the Angelman syndrome, 2 to 3 month old male and female *Ube3a* maternal deficient and knock-out mice were used. Mice (*Ube3a^m^^−/p+^*) with maternal hereditary *Ube3a* deficiency presented deficits in fluid consumption behavior, unlike the *Ube3a^m^^−/p^^−^* mice. Moreover, dysphagia was confirmed in *Ube3a* maternal deficient and knock-out mice, which showed a prolonged interval between licks and increased lick frequency in *Ube3a^m^^−/p^^−^* and *Ube3a^m^^−/p+^* mice. 

Dysphagia was confirmed in *Braf*^Q241R/+^ mice, the animal model of cardio-facio-cutaneous syndrome, through anatomical and histopathological analysis [42]. *Braf*^Q241R/+^ mice presented disturbance of feeding in newborns and disorders in esophageal dilatation. Dysphagia was verified as decreased contraction of esophageal tissue and histological alterations in the forestomach. 

Several articles evaluated the possibility of dysphagia due to lack of coordination between swallowing and ventilation in the animal model of respiratory failure [43,44]. In both of the two retrieved articles, 250–300 g male rats were used. Barometric plethysmography showed that swallowing during inhalation was increased in the hypercapnia. It was claimed that this can explain the possibility of aspiration during respiratory failure [43]. In another article, the swallowing frequency was remarkably high in rats with pulmonary emphysema compared to the control group [44]. The frequency of swallowing during exhalation was decreased in rats with pulmonary emphysema and pulmonary fibrosis. This suggested that respiratory diseases are related to swallowing–ventilation coordination and may increase risks of aspiration [44]. 

Dysphagia can occur in a Rdh10 mutant mouse embryo with defects in morphogenesis of pharyngeal skeletal elements and routing of pharyngeal motor nerves [45]. Dysphagia demonstrated as fetal mouth movement defects correlated with cleft palate in Rdh10 was confirmed through X-ray microtomography, in utero ultrasound video, ex vivo culture, and tissue staining. 

An article confirmed the effect of serotonin (5-hydroxytryptamine [5-HT]) deficiency on swallowing function [46]. In VFSS analysis, 5-HT deficiency had an impact on all three phases of swallowing. Dysphagia was confirmed because the TPH2^-/-^ mice had significantly slower licking and swallowing rates, and faster esophageal transit times, compared to the control groups. 

A neuro-degenerative disorder model that causes dysphagia was also reported [47]. Mutant mice with loss of Uch-L1 and Uch-L3 showed an early fatality rate because of dysphagia. Histopathological analysis showed that axonal degeneration was seen in nucleus tractus solitaires and area postrema of the medulla, which is necessary for central nervous system control for swallowing.

## 4. Discussion

### 4.1. Oropharyngeal Dysphagia

Dysphagia can be divided into three major phases: oral, pharyngeal, and esophageal. The oral and pharyngeal phases are generally classified together in the clinic as oropharyngeal dysphagia [48]. The oral phase is a voluntary process where food bolus is formed and transferred from the mouth to the pharynx. Dysphagia in the oral phase means that there is a problem with this process. Oral phase dysphagia usually results from impaired control of tongue, cheek, tooth, and oral floor muscles. It can result in poor bolus formation and management, such as difficulty chewing food, inability to keep the bolus in the mouth, difficulty gathering the bolus at the base of tongue, and hesitation to initiate swallowing [49,50]. As a result, it negatively influences the pharyngeal phase. Swallowing in the pharyngeal phase is an involuntary process that includes the swallowing reflex ranging from the pharynx to the esophagus. Dysphagia in the pharyngeal phase refers to a problem in the swallowing reflex or a sense of discomfort in the muscle involved in passing the food bolus from the pharynx to the esophagus. The pharyngeal phase can be negatively impacted by poor movement of tongue base, inadequate pharyngeal constriction, decreased hyolaryngeal excursion, etc. Any of these issues can result in penetration/aspiration and can also impact the esophageal phase [50]. Oropharyngeal dysphagia is generally attributed to neurological causes that lead to injuries (e.g., ischemic, traumatic, neoplastic, neurodegenerative, and infectious) in the nervous system that initiate and control swallowing (e.g., basal ganglia, brain stem, cerebellum, and cranial nerve) [50,51,52]. Neurologic diseases include strokes, brain tumors, movement disorders such as PD and HD, MS, cognitive impairments, and motor neuron diseases of the cerebellum and the brain stem such as ALS. Congenital malformations, such as cerebral palsy and cleft lip and palate, can cause dysphagia. Oropharyngeal dysphagia can occur due to other causes. The muscles used to swallow can be weakened with age. This can explain why swallowing difficulties are relatively common in the elderly [48]. Reparatory failure that makes it difficult to breathe properly affects swallowing ability. Moreover, dysphagia can develop as a complication of head and neck surgery or radiation treatment. The esophageal phase is more associated with the intrinsic nerve distribution than the central nerve system. Esophageal dysphagia develops when there are pathological problems with the esophagus. Esophageal dysphagia was not in the scope of this review, which focused only on oropharyngeal dysphagia. When symptoms of dysphagia are presented, prompt examinations to confirm the exact organic causes and appropriate therapeutic strategies are required. If structural issues are present and result in dysphagia, then removal of those obstructions (tethers, tumors, etc.) can result in damage to nerves, glands, and muscles, thereby exacerbating swallowing deficits. However, for the improvement of oropharyngeal dysphagia, appropriate treatments of neurological or muscular diseases that cause dysphagia are essential but absent. For the fundamental treatment of oropharyngeal dysphagia, the exact causes should be analyzed, and treatment and countermeasures should be accordingly suggested. There is no fundamental treatment for dysphagia yet. In addition, there is no definite means to prevent dysphagia in advance due to its variety of causes. Therefore, the requirement to develop the most effective and appropriate therapeutic strategies according to the causes of dysphagia has been emphasized. Research on oropharyngeal dysphagia using animal models has shown considerable progress in understanding its pathologic physiology in recent years. Thus, this systematic literature review attempts to provide a basis for developing therapeutic strategies by gathering and presenting information related to rodent oropharyngeal dysphagia models with various causes such as neurodegenerative disease, neuromuscular disease, congenital malformation, and respiratory failure. In this systematic review, we screened 1525 publications and summarized 37 original articles of rodent disease models of oropharyngeal dysphagia. We provide a summary of the evaluation data of dysphagia used in these disease models to help define the current state of the field of rodent pre-clinical dysphagia models. This review will provide insights for future research by summarizing the characteristics of rodent models of oropharyngeal dysphagia. Moreover, we believe that our review can help researchers studying dysphagia to select an appropriate pre-clinical animal model.

### 4.2. Disease Rodent Models with Oropharyngeal Dysphagia

Characteristics of oropharyngeal dysphagia were verified in various disease rodent models. Among the articles on disease models, most reported dysphagia symptoms in rodent models of PD, followed by stroke, ALS, DiGeorge(22q11) deletion syndrome, and DS. Among the various rodent models used for PD study, dysphagia was confirmed in the classical 6-hydroxydopamine (6-OHDA) rat model, in which the dopamine nigro-striatal pathway is destroyed; the rotenone model; another toxin-based model, in which degeneration of the nigro-striatal tract was induced by systematically injecting rotenone; and the PTEN-induced putative kinase (PINK 1) knockout rat (PINK^-/-^) [11,12,13,14,15,16,17,18,53]. The 6-OHDA rat model was most common, followed by PINK1^-/-^ rat and rotenone infusion models. Rats were used in all of the three PD rodent models. It is surmised that rats were used more than mice because the 6-OHDA model needs to be injected in a small brain region such as the medial forebrain bundle, and because the region of interest was evaluated to develop toxin-based models of PD [54]. In addition, the recent growth of transgenic techniques enabled using more rats as a genetic PD animal model [55]. PD is a complicated neurodegenerative disease that affects cranial sensorimotor functions such as speech and swallowing. The PD rat models described in Table 1 confirmed dysphagia by showing vocalization, oromotor, and swallowing deficits (Table 1). Dysphagia was evaluated in the rat model of middle cerebral artery occlusion (MACO), which is one of the most common cerebral ischemia experimental animal models of the stroke. Through evaluations such as body weight measurement, histological analysis, VFSS, and electrocardiogram in unilateral transient MACO models, in which ischemia is induced by monofilament for 90 min, possible use of the MACO rat model as a stroke-induced dysphagia animal model was confirmed [19,20,21]. In the chronic cerebral hypoperfusion model, which was induced by permanent ligation of bilateral common carotid arteries in rats, aspiration was confirmed through abnormal swallowing reflex and bacteria culture from the lung [22,23,24]. By checking feeding problems through body weight measurement in neonatal hypoxia ischemia (HI) model induced by unilateral common carotid artery cauterization, dysphagia was also implied in mice with cerebral infarcts [25]. Among ALS rodent models, superoxide dismutase 1 (SOD1)-G93A transgenic mice or rat models were reported to have dysphagia. ALS-related dysphagia was mimicked by intralingual CTB-SAP injection. It was suggested that the SOD1-G93A transgenic mouse or rat model is a suitable animal model for oral and pharyngeal dysphagia in ALS, showing oromotor deficit, pharyngeal impairment, change in swallowing pattern, and tissue finding of neurodegeneration related to swallowing [26,27,28]. Osman et al. focused on the two types of transgenic SOD1 mice model of ALS: the widely used high copy number (HCN) SOD1-G93A model; and the rarely used low copy number (LCN) SOD1-G93A model [56,57]. HCN-SOD1 model expresses 25 copies or less of the mutant transgene, and gradually develops paralysis in the hind limbs from 3 months old and dies around at 4 to 5 months old [56,57,58,59]. The LCN-SOD1 model expresses eight or fewer copies of the mutant transgene [60,61], which delays clinical outbreak of hind limb disorder (within 6 to 7 months) and extends lifetime (7 to 10 months) [61,62,63]. Osman et al. aimed at comparing dysphasia phenotypes in LCN and HCN transgenic SOD1 mouse models of ALS. They suggested using the LCN-SOD1 mouse model that emulated clinical pathological features of swallowing disorders in an advanced stage of their ALS facilitates understanding of the mechanisms contributing to swallowing disorders in ALS [29]. In the early disease stage of the SOD1-G93A rat model, orolingual defects such as tongue motility deficit are presented; however, limb muscles are reported to be more affected than muscles in the head and neck [30]. Lind et al. suggested that this new and inducible model, exhibiting death of hypoglossal motor neurons by injecting CTB-SAP, reproduces the dysphagia phenotype observed in ALS rodent models [31]. Many animal models induced OPMD in rodents, but most of these failed to evaluate dysphagia, so were excluded from this systematic review [64,65,66]. In one analyzed article, A17 PABPN1 transgenic mice were used as OPMD animal models to induce dysphagic disease [32]. In addition, the LgDel mouse model of chromosome 22q11 deletion syndrome showed a change in feeding and swallowing accompanied by craniofacial morphological abnormality, neuroformation disorder, and lung infections, which are essential for feeding and swallowing [33,34,35]. This suggests that the LgDel mouse model can be used as a rodent model of pediatric dysphagia. Ts65Dn and Dp(16)1Yey mice were used as the DS animal model to confirm dysphagia. Dysphagia was verified by proposing biochemical differences in parts (not all) of muscles related to swallowing and mandibular movement in Ts65Dn mice [36,37,38]. Both Ts65Dn and Dp(16)1Yey mice showed differences in body weight, mastication rate, and anterior digastric muscle size compared to the control group. However, the Ts65Dn model was suggested to be a suitable animal model for study of dysphagia related to DS in a report that the Ts65Dn model of DS is more affected than other genotype groups. For the Huntington’s disease (HD) animal model, transgenic mice, from which D1 dopamine receptor (*Drd1a*)-expressing cells are gradually removed from the postnatal brain, were developed and showed oral behavior disorders such as chewing [39]. HLA-DRB1*0401-restricted MBP 111–129-specific Humanized TCP transgenic mice emulating multiple sclerosis (MS) presented dysphagia induced by restriction in mandible and tongue movement [40]. The *Ube3a* deficiency mouse was used as the animal model of Angelman syndrome, and a behavioral defect in fluid consuming activity was seen in maternally inherited *Ube3a* deficiency (*Ube3a*^m−/p+^). Oropharyngeal dysphagia was indirectly confirmed from the slower lick rhythm than that of the wild type [41]. Knock-in mice (*Braf*^Q241R/+^) expressing *Braf Q241R* mutation, which presents a phenotype related to cardio-facio-cutaneous syndrome, were reported to have difficulty in neonatal feeding and esophageal dilatation [42]. Aspiration may occur during respiratory failure from lack of coordination between swallowing and ventilation. Increased risk in aspiration was reported in rats with experimentally induced pulmonary emphysema and pulmonary fibrosis [43,44]. Additionally, probability of dysphagia was also identified in Rdh10 mutant mice with cleft palate due to retinoid deficiency, TPH2 knockout (TPH^-/-^) mice with serotonin (5-hydroxytryptamine [5-HT]) deficiency, and Uch-L1^gad^/Uch-L3^Δ^^3–7^ double homozygous mice used as a neuro-degenerative animal model [45,46,47].

### 4.3. Limitation and Future Direction

This review focuses only on oropharyngeal dysphagia according to criteria set by the authors. It is considered that further studies providing information on esophageal dysphagia are necessary for a comprehensive understanding of dysphagia. This review lists only rodent animal models. Rodents, similar to humans, take nutrition through nursing in the early phase after birth, and gradually develop the ability to chew and swallow solid food. Therefore, although the restriction of this study to rodent animal models is a limitation, it is clear that the rodent is a suitable animal model for study of dysphagia. Nonetheless, careful consideration should be given to the extent to which various rodent models presenting dysphagia can reproduce the state of dysphagia in humans, and how successfully the results of the studies using these models can be interpreted. Although dysphagia studies related to aging have been reported as the aged population increases, this review, according to the selection criteria, excluded rodent animal models with dysphagia that is simply presented because of aging. To improve dysphagia symptoms caused by aging, research on animal models related to this factor is necessary in the future. Moreover, a systematic review suggesting the criteria for animal models with dysphagia related to aging would be needed. Finally, quality assessment of the collected articles was not performed in this systematic review because the aim was to introduce rodent models of oropharyngeal dysphagia, and to propose methods used to evaluate dysphagia in these models and their results. Despite its limitations, this systematic review can be meaningful to researchers because it provides an up-to-date and systematically organized overview of the available rodent models of oropharyngeal dysphagia.

## 5. Conclusions

We collected 37 articles to analyze various disease and injury models of oropharyngeal dysphagia developed to date. The disease animal models, damage protocols, animals used, and the results of dysphagia assessment from the retrieved articles were summarized. We believe that this review will be of help in selecting appropriate animal models for studies of dysphagia.

## Figures and Tables

**Table 1:** Characterization of oropharyngeal dysphagia in rodent models of Parkinson’s disease.

Author (Year)	Animal/Sex, Age or BW	Method Inducing Dysphagia	Main Defects	Dysphagia Screening	
				Methods (Tools)	Results
Ciucci et al. (2010) [11]	Rat/male, 4–6 months old	Unilateral infusion of 6-OHDA into the medial forebrain bundle	Degeneration of dopamine neurons; vocalization deficits	· Ultrasonic vocalization analysis	· The complexity, intensity and bandwidth diminish in unilateral 6-OHDA model.
Ciucci et al. (2011) [12]	Rat/male, 9 months old	Unilateral infusion of 6-OHDA into the medial forebrain bundle	Degeneration of dopamine neurons	· Tongue motility assessment	· Maximal and average tongue force was significantly diminished and average tongue press time was significantly longer after unilateral infusion of 6-OHDA.
Kane et al. (2011) [13]	Rat	Unilateral infusion of 6-OHDA into the medial forebrain bundle	Degeneration of presynaptic dopaminergic striatal neurons	· Ultrasonic vocalizations analysis · Pasta biting	· The parkinsonian animals have markedly lower bite strength and irregular intervals between bites. · The parkinsonian animals took about twice as long to consume a single piece of pasta as the control animals.
Nuckolls et al. (2012) [14]	Rat/male, 3 months old	Unilateral or bilateral infusion of 6-OHDA into the medial forebrain bundle	Depletion of dopamine	· Lick assay	· Both unilateral and bilateral nigrostriatal dopamine depletion decrease tongue force during licking. · Tongue motility is decreased following unilateral but not bilateral nigrostriatal dopamine depletion.
Russell et al. (2013) [15]	Rat/male, 9 months old	Unilateral infusion of 6-OHDA into the medial forebrain bundle	Degeneration of presynaptic dopaminergic striatal neurons	· VFSS	· Parkinsonian group had significantly more aberrant movement than the healthy young adult and old groups. · Parkinsonian group had significantly smaller bolus areas compared with both the young adult and old groups.
Grant et al. (2015) [16]	Rat/2, 4, 6, and 8 months old	Mutations to the Pink1 (PINK1^-/-^ rats)	Progressive vocalization and oromotor deficits	· Ultrasonic vocalization analysis · Lick force and rate · Pasta biting	PINK1^-/-^ rats · Developed early and progressive vocalization and oromotor deficits. · Had significantly reduced loudness (intensity) as early as 2 months of age. · Had significantly reduced peak frequency at 6 and 8 months compared to both 2 and 4 months. · Pressed with a greater amount of force compared to controls during the licking task. In addition to overshooting, the target PINK1^-/-^ rats also had more variable lick forces compared to controls. As the PINK1^-/-^ rats aged, their lick force pattern changed. · Had more irregular and inconsistent biting patterns leading to increased inter-bite intervals.
Cullen et al. (2018) [17]	Rat/male, 4 months old	Mutations to the Pink1 (PINK1^-/-^ rats)	Progressive vocalization and oromotor deficits	· VFSS	Pink1^-/-^ rats · Had significantly increased the average and maximum bolus size at both 4 and 8 months. · Had significantly increased velocities compared to WT at 8 months. · Had a significant reduction in mastication rate for at 8 months.
Gould et al. (2018) [18]	Rat/male and female, 14 weeks old	Daily IP injections of the rotenone emulsification	Debilitative behaviors (akinesia and lack of feeding)	· VFSS	· A significant effect of injection with rotenone, regardless of dose level, was found for rostrocaudal mandibular range of motion, duration of chewing cycle, duration of jaw closing, and time of tongue rostralmost movement.

6-OHDA, 6-hydroxydopamine; BW, body weight; IP, intraperitoneal injection; PINK1, PTEN-induced putative kinase 1; VFSS, videofluoroscopic swallowing study; WT, wild type.

**Table 2:** Characterization of oropharyngeal dysphagia in rodent models of stroke.

Author (Year)	Animal/Sex, Age or BW	Method Inducing Dysphagia	Main Defects	Dysphagia Screening	
				Methods (Tools)	Results
Dittmar et al. (2003) [19]	Rat/male, 250–300 g	Unilateral MCAO	Focal cerebral ischemia(ischemic changes in the right mastication muscles and pharynx)	· MRI · BW measurement · Histological assessment	· In 49% of MCAO group, ischemic tissue damage to the ipsilateral ECA area, including temporal, lingual, and pharyngeal musculature, was detectable by MRI. · Histology of temporal muscles confirmed acute ischemic myopathy. · Animals with ECA territory ischemia showed delayed BW development and poorer recovery of motor function.
Cullins and Connor (2019) [20]	Rat/male, 6 weeks old	Unilateral MCAO	Reductions in tongue protrusion and licking efficiency	· Tongue force · VFSS	· Maximum voluntary tongue force, bolus area, and bolus speed were significantly reduced in the MCAO group at 1 and 8 weeks.
Sugiyama et al. (2014) [21]	Rat/male, 8 weeks old, 270–310 g	Unilateral MCAO	Pharyngeal-stage swallowing disorders	· Electromyogram (swallowing reflex)	· Two weeks after MCAO, the number of swallows significantly decreased and the onset latency of the first swallow was prolonged compared with that of the sham group.
Ikeda et al. (2015) [22]	Rat/male spontaneous hypertensive rats, 18–19 weeks old, 326–392 g)	BCAO	Chronic cerebral hypoperfusion (disorders of the swallowing reflex)	· Swallowing reflex	· The swallowing reflex gradually decreased after BCAO.
Zhang et al. (2009) [23]	Rat/male, 8 weeks old)	LBCCA	Chronic cerebral hypoperfusion	· Swallowing reflex analysis · Bacteria culture	· LBCCA caused a significantly longer latency to swallowing. · Bacterial colonies were found in lung cultures of the vehicle-treated group after 14 days of LBCCA; the numbers of bacterial colonies increased with time.
Asano et al. (2017) [24]	Rat/male, 10 weeks old	LBCCA	Dysfunction of the swallowing reflex	· Visual observation of the laryngeal movement	· Ligation of bilateral common carotid arteries caused BW loss and a decrease in survival rates in the rats. · Ligation of bilateral common carotid arteries markedly attenuated the swallowing responses.
Bellot et al. (2014) [25]	Mouse/both genders, p7	Cauterization of unilateral common carotid artery	Cerebral infarcts	· BW and behavior measurement · Plethysmography · HPLC	· Mice with cerebral infarct expressed motor disturbances and had a lower BW and a decreased respiratory frequency than sham. · 5-HT, HT, noradrenaline, and dopamine contents were increased in both the medulla and the spinal cord of mice with cerebral infarcts.

BCAO, bilateral common carotid arteries occlusion; BW, body weight; ECA, external carotid artery; HPLC, high-performance liquid chromatography; LBCCA, ligation of bilateral common carotid artery; MCAO, middle cerebral artery occlusion; MRI, magnetic resonance imaging; VFSS, videofluoroscopic swallowing study.

**Table 3:** Characterization of oropharyngeal dysphagia in rodent models of amyotrophic lateral sclerosis.

Author (Year)	Animal/Sex, Age or BW	Method Inducing Dysphagia	Main Defects	Dysphagia Screening	
				Methods (Tools)	Results
Lever et al. (2009) [26]	Mouse/male and female, 6 weeks old	SOD1-G93A transgenic mice	Dysphagia in a manner similar to human with ALS	· Oral behavioral assessment (lick and mastication rates) · BW and food and water consumption · Histological analysis	· At asymptomatic (60 days), disease onset (110 days), and disease end stage (140 days), lick and mastication rates were significantly lower for transgenic mice compared with controls. · WT and transgenic mice consumed essentially the same amount of food over time; food consumption was more variable over time for transgenic than for WT mice; water intake for transgenic mice significantly declined over time. No significant differences were observed between the mean food/or water consumption of WT and transgenic animals at each time point. · Histologic analysis of the brainstem showed marked neurodegeneration (vacuolation) of the trigeminal and hypoglossal nuclei.
Lever et al. (2010) [27]	Mouse/male and female, 7 weeks old)	SOD1-G93A transgenic mice	Dysphagia in a manner similar to human with ALS	· Electrophysiological and histological analysis	· Transgenic mice required a two-fold higher stimulus frequency (40 Hz) applied to the SLN to evoke swallowing compared with WT controls (20 Hz); transgenic females required a significantly higher stimulus frequency applied to the SLN to evoke swallowing compared with transgenic males. · Histological evidence of neurodegeneration (vacuoles) was identified throughout representative motor (nucleus ambiguus) and sensory (nucleus tractus solitarius) components of the pharyngeal stage of swallowing.
Shock et al. (2015) [28]	Mouse/4–8 months old	SOD1-G93A transgenic mice	LAR impairment	· Laryngoscopy analysis	· VF abduction phase duration and mean LAR duration was shorter for ALS-affected mice compared to controls; however, statistical significance was not achieved.
Osman et al. (2019) [29]	Mouse/male and female	High copy number (HCN) SOD1-G93A and low copy number (LCN) SOD1-G93A model	HCN SOD1-G93A: a tongue motility deficit LCN SOD1-G93A: lengthening of the asymptomatic phase and disease duration	· VSFF · Postmortem assays	· Lick rate, swallow rate, inter-swallow interval, and pharyngeal transit time were significantly altered in both HCN-SOD1 and LCN-SOD1 mice compared to controls. · Tongue weight, tongue dorsum surface area, total tongue length, and caudal tongue length were significantly reduced only in the LCN-SOD1 mice compared to age-matched controls. · LCN-SOD1 mice with lower BW had smaller/lighter weight tongues, and those with forelimb paralysis and slower lick rates died at a younger age. · LCN-SOD1 mice had a 32% loss of hypoglossal neurons, which differed significantly when compared to age-matched control mice.
Smittkamp et al. (2010) [30]	Rat/male and female	SOD1-G93A rat	Bulblar motor deficits	· Behavioral assessment (orolingual motor function)	· A persistent tongue motility deficit appeared in the early phase * of the disease. · No differences in tongue force between control and SOD1-G93A rats, demonstrating that tongue muscle strength was maintained in SOD1-G93A rats.
Lind et al. (2018) [31]	Rat/male, 3–4 months old	Intralingual injections of CTB-SAP into the genioglossus muscle	Death of hypoglossal motor neurons	· Immunohistochemistry (hypoglossal motor neuron survival) · Nerve recording (hypoglossal motor output) · VFSS (swallowing and licking)	· CTB-SAP treated rats exhibited targeted hypoglossal motor neuron death; decreased hypoglossal motor output; and swallowing and lick deficits.

ALS, amyotrophic lateral sclerosis; BW, body weight; CTB–SAP, cholera toxin B conjugated to saporin; LAR, laryngeal adductor reflex; SLN, superior laryngeal nerve; SOD 1, superoxide dismutase 1; VF, vocal fold; VFSS, videofluoroscopic swallowing study; WT, wild type. * They tested at early (maturation), middle (pre-symptomatic), and late (symptomatic and end-stage) phases of the disease.

**Table 4:** Characterization of oropharyngeal dysphagia in rodent models of other diseases.

Disease Model	Author (Year)	Animal/Sex, Age or BW	Method Inducing Dysphagia	Main Defects	Dysphagia Screening	
					Methods (Tools)	Results
**OPMD**	Randolph et al. (2014) [32]	Mouse/male and female, various ages	A17 PABPN1 transgenic mice	Myopathologic features similar to those of OPMD patients	· Lick assay · Histological analysis	· Overexpression of mutant A17 PABPN1 differentially affected growth of the palatopharyngeus muscle dependent on its location within the pharynx. · Lick rates of A17-MUT mice were significantly decreased with age and impaired compared to A10-WT mice.
**DiGeorge (22q11) deletion syndrome**	Karpinski et al. (2014) [33]	Mouse/P1–30 pups	LgDel mouse	Dysphagic symptoms, craniofacial changes, alteration of CNs development	· BW measurement · Histological analysis	· LgDel pups gain significantly less weight during the first postnatal weeks and have several signs of respiratory infections due to food aspiration. · Palate and jaw anomalies indicate divergent oro-facial morphogenesis. · Gene expression, axon growth and sensory ganglion formation in the trigeminal (V), glossopharyngeal (IX), or vagus (X) CNs that innervate targets essential for feeding, swallowing, and digestion are disrupted.
	Wang et al. (2017) [34]	Mouse/P7–14 pups	LgDel mouse (inherited paternally)	Cognitive impairments, feeding and swallowing difficulties	· Electrophysiological analysis	· The after-hyperpolarization potential is significantly shorter in duration and greater in magnitude in LgDel mouse pups. · Glutamate amplitudes in hypoglossal neurons are diminished in LgDel siblings. · GABAergic events are less frequent in hypoglossal motor neurons in LgDel animals.
	Welby et al. (2020) [35]	Mouse/male and female, 3 months old	LgDel mouse (inherited paternally)	Dysphagia	· Fluoroscopic and endoscopic assessments · Video surveillance · Postmortem assessment	· Transoral endoscopic assessment identified minor structural anomalies of the palate and larynx in one-third of the LgDel mice examined. · Video surveillance of feeding-related behaviors showed that LgDel mice eat and drink more frequently. · LgDel mice have significantly increased lung inflammation, a potential sign of aspiration-based dysphagia.
**Down syndrome**	Glass and Connor (2016) [36]	Mouse/male and female, P11 and 5–6 and 10–53 weeks old	Ts65Dn mouse	Cognitive impairment	· SDS-PAGE, immunofluorescence, and qRT-PCR · VSFF and mastication assays (Functional feeding and swallowing performance)	· Both the anterior and posterior digastric muscles in 11 day and 5–6 week old Ts65Dn groups showed significantly lower MyHC 2b protein levels than in age-matched euploid control groups. · 10–11 week old Ts65Dn mice revealed significantly less MyHC 2b mRNA expression in the posterior digastric, but not the anterior digastric muscle as compared with euploid controls. · 10–53 week old Ts65Dn mice revealed lower levels of MyHC 2b protein in the posterior digastric muscles of Ts65Dn than in euploids, but similar levels of MyHC 2b in the anterior digastric muscles. · 5–6 week old Ts65Dn and euploid controls showed similar swallow rates, inter-swallow intervals, and mastication rates.
	Glass et al. (2019) [37]	Mouse/male, 5 weeks old	Ts65Dn and Dp(16)1Yey mouse	□-	· BW measurement · Licking behavior and mastication assays · Measurement of digastric muscle size	· Genotype was associated with significant differences in weight gain, mastication rate, and anterior digastric muscle size, and significant reductions in size of anterior digastric myofibers positive for MyHC 2a. · Ts65Dn model of DS was more affected than other genotype groups.
	Glass et al. (2019) [38]	Mouse/male and female, 8–44 weeks old	Ts65Dn and Dp(16)1Yey mouse	□-	· VFSS · Immunofluorescence, SDS-PAGE	· Adult Ts65Dn showed significantly slower swallow rates, longer inter-swallow intervals, and greater numbers of jaw excursion cycles preceding each swallow. · Adult Dp(16)1Yey mice showed swallowing performance similar to control mice. Exploratory quantitative analyses of the intrinsic tongue, and extrinsic tongue muscles showed no significant differences between genotype groups in MyHC isoform profiles.
**Huntington’s disease**	Gantois et al. (2007) [39]	Mouse/male and female, 4–18 weeks old	A transgenic mouse in which Drd1a+ cells are slowly but progressively ablated in the postnatal striatum, yet Drd2 single-positive cells are preserved.	Major abnormalities in oral functions of sifting and chewing	· Oral behavioral analysis(chewing)	· At 6–9 weeks, mutant mice showed a decrease in chewing in both sexes.
**Multiple sclerosis**	Quandt et al. (2004) [40]	Human TCR-HLA-DRB1*0401–IA^-/-^ Tg Mice	HLA-DRB1*0401-restricted MBP 111–129-specific Humanized TCR Transgenic Mice	Swallowing and lingual paralysis	· Clinical features · Histological examination	· Mice displayed dysphagia due to restriction in jaw and tongue movements and abnormal gait.
**Angelman syndrome**	Heck et al. (2008) [41]	Mouse/male and female, 2–3 months old	*Ube3a^m−/p−^* and *Ube3a^m−/p+^* maternal deficient and knock-out mice	Abnormal cerebellar neuronal activity	· Fluid consumption and licking behavior	· Mice with a maternally inherited *Ube3a* deficiency (*Ube3a^m−/p+^*) show defects in fluid consumption behavior which are different from *Ube3a^m−/p−^* mice. · *Ube3a^m−/p−^* and *Ube3a^m−/p+^* mice exhibit an extended inter-lick interval and increased frequency of licks per visit.
**Cardio-facio-cutaneous syndrome**	Inoue et al. (2017) [42]	Mouse	knock-in mice expressing a *Braf Q241R* mutation	Growth retardation, craniofacial dysmorphisms, congenital heart defects	· Anatomical and histological analysis	· *Braf*^Q241R/+^ mice have neonatal feeding difficulties and esophageal dilation. · The esophagus tissues from *Braf*^Q241R/+^ mice displayed incomplete replacement of smooth muscle with skeletal muscle and decreased contraction. · *Braf*^Q241R/+^ mice showed hyperkeratosis and a thickened muscle layer in the forestomach.
**Respiratory failure**	Ghannouchi et al. (2013) [43]	Rat/male 250–350 g	At different gas concentration (hypercapnia, hypoxia, and tachypnea	Respiratory failure explained by a lack of co-ordination between swallowing and ventilation	· Barometric plethysmograph	· Hypercapnia increased swallowing during inspiration, which was not the case for tachypnea or hypoxia, and could explain some aspirations during respiratory failure.
	Ghannouchi et al. (2019) [44]	Rat/male, 2–3 months, 250–300 g	Fibrosis: an intratracheal instillation of bleomycin, emphysema: a single intratracheal instillation of porcine pancreatic elastase	Pulmonary fibrosis and pulmonary emphysema	· Barometric plethysmograph	· Compared to control groups, swallowing frequency was significantly higher in rats with pulmonary emphysema. · There was a decrease in swallows during expiration in rats with pulmonary emphysema and in rats with pulmonary fibrosis.
**Cleft palate**	Friedl et al. (2019) [45]	Mouse/embryo	Rdh10 mutant	Defects in morphogenesis of pharyngeal skeletal elements and routing of pharyngeal motor nerves	· X-ray microtomography · in utero ultrasound video · ex vivo culture and tissue staining	· Mouse embryos deficient in retinoic acid have mispatterned pharyngeal nerves and skeletal elements that block spontaneous fetal mouth movement in utero.
**5-HT deficiency**	Haney et al. (2019) [46]	Mouse/male and female, 6 (young) and 12 (older) months old	TPH2^-/-^ mice (mice deficient in TPH2)	Swallowing disorders	· VFSS	· 5-HT deficiency altered all three stages of swallowing. Compared with controls, TPH2^-/-^ mice had significantly slower lick and swallow rates, and faster esophageal transit times.
**Neuro-degenerative disorders that cause dysphagia**	Kurihara et al. (2001) [47]	Mouse/(Uch-L1^gad^/Uch-L3^∆^^3−7^ double homozygous)	Mutant mice (loss of Uch-L1 and Uch-L3)	Dysphagia, neurodegeneration, posterior paralysis	· Food intake assay · Histological analysis	· The double homozygous weight was 30% less than that of single homozygotes and displayed an earlier onset of lethality, possibly due to dysphagia, a progressive loss in the ability to swallow food. · Histological analysis revealed that axonal degeneration of the nucleus tractus solitaires ^‡^ and area postrema of the medulla.

5-HT, 5-hydroxytryptamine; BW, body weight; CN, cranial nerve; DS, Down syndrome; GABA, γ-aminobutyric acid; MyHC, myosin heavy chain; OPMD, oculopharyngeal muscular dystrophy; PABPN1, polyadenylate-binding nuclear protein 1; qRT-PCR, quantitative real time polymerase chain reaction; SDS-PAGE, sodium dodecyl sulphate–polyacrylamide gel electrophoresis; TPH2, tryptophan-hydroxylase-2; VFSS, videofluoroscopic swallowing study; WT, wild type. ^‡^ nucleus tractus solitaires is essential for central nervous system control of swallowing.

## Data Availability

Not applicable.
